# Influence of Added Cellulose Nanocrystals on the Rheology of Polymers

**DOI:** 10.3390/nano15020095

**Published:** 2025-01-09

**Authors:** Rajinder Pal, Parth Deshpande, Smit Patel

**Affiliations:** Department of Chemical Engineering, University of Waterloo, Waterloo, ON N2L 3G1, Canada; pydeshpande@uwaterloo.ca (P.D.); smithpatel19991@gmail.com (S.P.)

**Keywords:** cellulose nanocrystals, polymers, rheology, non-Newtonian, shear-thinning, viscosity, nanocrystal–polymer interaction

## Abstract

The interactions between cellulose nanocrystals and six different polymers (three anionic, two non-ionic, and one cationic) were investigated using rheological measurements of aqueous solutions of nanocrystals and polymers. The experimental viscosity data could be described adequately by a power-law model. The variations in power-law parameters (consistency index and flow behavior index) with concentrations of nanocrystals and polymers were determined for different combinations of nanocrystals and polymers. The interactions between nanocrystals and the following polymers: anionic sodium carboxymethyl cellulose and non-ionic guar gum, were found to be strong in that the consistency index increased substantially with the addition of nanocrystals to polymer solutions. The interaction between nanocrystals and non-ionic polymer polyethylene oxide was moderate. Depending on the concentrations of nanocrystals and polymer, the consistency index both increased and decreased upon the addition of nanocrystals to polymer solution. The interactions between nanocrystals and the following polymers: anionic xanthan gum, anionic polyacrylamide, and cationic quaternary ammonium salt of hydroxyethyl cellulose, were found to be weak. The changes in rheological properties with nanocrystal addition to these polymer solutions were found to be small or negligible.

## 1. Introduction

In many practical formulations of dispersed systems such as emulsions, suspensions, and foams, high molecular weight polymers are added to the matrix phase to improve the quality and shelf life of the product [1,2,3,4,5,6]. In some applications, polymers are used as thickeners to modify the rheology of the continuous phase and to impart high viscosity and yield strength on that phase. The thickening effect causes the inclusions of the dispersed phase (droplets, particles, bubbles) to remain suspended in the continuous phase. Consequently, sedimentation/creaming of inclusions of emulsions, suspensions, and foams is weakened or even eliminated and the shelf life of the product is extended. In food products consisting of emulsions, suspensions, and foams, the additional reason for adding polymeric thickeners (mainly polysaccharides) to the continuous phase is to impart the required mouth-feel properties, pleasing appearance, and texture to the food products and to improve their moisture retention. Some examples of polymeric thickening agents used widely in the formulations of dispersed systems are sodium carboxymethyl cellulose (CMC), hydroxyethyl cellulose, xanthan gum, guar gum, gum tragacanth, gum karaya, gum Arabic, locust bean gum, and polyacrylamides. The rheology and thickening effect of polymeric agents is well studied in the literature [7,8,9,10,11,12,13,14,15,16,17,18].

In order to improve the consistency, quality, and shelf life of the dispersed systems, non-polymeric materials such as nanoclays and nanoparticles are also often used [19,20,21,22]. The addition of nanoclays and nanoparticles to the continuous phase provides the necessary thickening effect. Recently, however, nanocrystalline cellulose (NCC) has gained significant attention as a rheology modifier and thickening agent due to its surface charge and high aspect ratio. Nanocrystalline cellulose (NCC) in the form of cellulose nanocrystals is a versatile cost-effective nanomaterial [23,24,25,26,27,28,29,30,31,32,33,34,35,36,37,38,39,40,41]. It is derived from cellulose, the most abundant organic polymer on earth sourced from wood, cotton, and agricultural residues. NCC is often produced through acid hydrolysis of cellulose fibers in the form of rod-shaped nanocrystals with diameters typically ranging from a few nanometers to 100 nm and lengths of up to several hundred nanometers. The nanocrystals of NCC exhibit a highly crystalline structure with glucose units aligned in parallel along their longitudinal axis. Consequently, NCC exhibits exceptional mechanical properties such as high tensile strength and stiffness. Due to its biodegradability and biocompatibility, NCC is an environmentally friendly alternative compared with petroleum-based materials. Furthermore, it is a sustainable material derived from renewable resources. Also, the surface chemistry of NCC allows for facile functionalization. Different functional groups could be attached to the surface of nanocrystals to tailor their properties for specific applications. This versatility extends their applicability to many industrial applications.

The broad objective of this work was to investigate the interactions between polymers and NCC with the goal of developing novel hybrid NCC-polymer thickeners and rheology modifiers. To that end, the rheology of mixtures of NCC and polymer was investigated over a broad range of concentrations of NCC and polymer. To our knowledge, this is the first study where the interactions between NCC and polymers are investigated and the corresponding thickening effect established using rheology.

Polymer solutions generally behave as non-Newtonian fluids in that their viscosity varies with the applied shear rate. Non-Newtonian fluids, in general, can be broadly classified into three groups provided that their behavior is inelastic and time independent. The three groups are: pseudoplastic or shear-thinning, dilatant or shear-thickening, and fluids with yield stress (Bingham plastic and yield pseudoplastic). The most commonly encountered behavior in polymer solutions is pseudoplastic or shear-thinning. The viscosity of polymer solutions decreases with the increase in shear rate. While the non-Newtonian behavior of polymer solutions is well established, the rheological characterization of dispersions of nanoparticles and nanocrystals has received attention only recently. Note that dispersions of nanoparticles/nanocrystals are also referred to as nanofluids in the literature. Nanofluids have found many practical applications in different fields [42,43,44,45,46,47].

Kinra and Pal [36], Shafiei-Sabet et al. [37], and Wu et al. [40] have investigated the rheological behavior of NCC dispersions. The nanocrystal dispersions are shear-thinning at NCC concentrations larger than about 1 wt%. The dispersions become more viscous and shear-thinning with the increase in nanocrystal concentration. Pal and Pal [20] investigated the rheology of dispersions of starch nanoparticles. The concentration of starch nanoparticles varied from 0 to 25 wt%. The starch nanoparticle dispersions were Newtonian in nature in that viscosity was constant, independent of shear rate. However, the viscosity increased substantially with the increase in nanoparticle concentration. The thickening effect of nanoparticles and nanocrystals on the rheology of emulsions and suspensions have also been investigated recently by Kinra and Pal [36], Pal and Pal [20], Ghanaatpishehsanaei and Pal [21], and Pal and Pattath [19]. As already noted, to our knowledge, there is no information available in the published literature on the thickening effect or rheology of hybrid NCC-polymer thickeners.

## 2. Materials and Methods

### 2.1. Materials

Six different polymers, three anionic (CMC, polyacrylamide, xanthan gum), two non-ionic (guar gum, polyethylene oxide), and one cationic (quaternary ammonium salt of hydroxyethyl cellulose) were investigated. Table 1 lists the polymers investigated. The chemical structure and uses of polymers are also summarized in the table.

The following three factors were considered in the selection of polymers for NCC–polymer mixture study: electric charge (anionic, cationic, non-ionic), thickening ability, and commercial availability. The polymers selected included all three types: anionic, cationic, and non-ionic. They could all provide a thickening effect, and they are available commercially.

**Figure 1:**
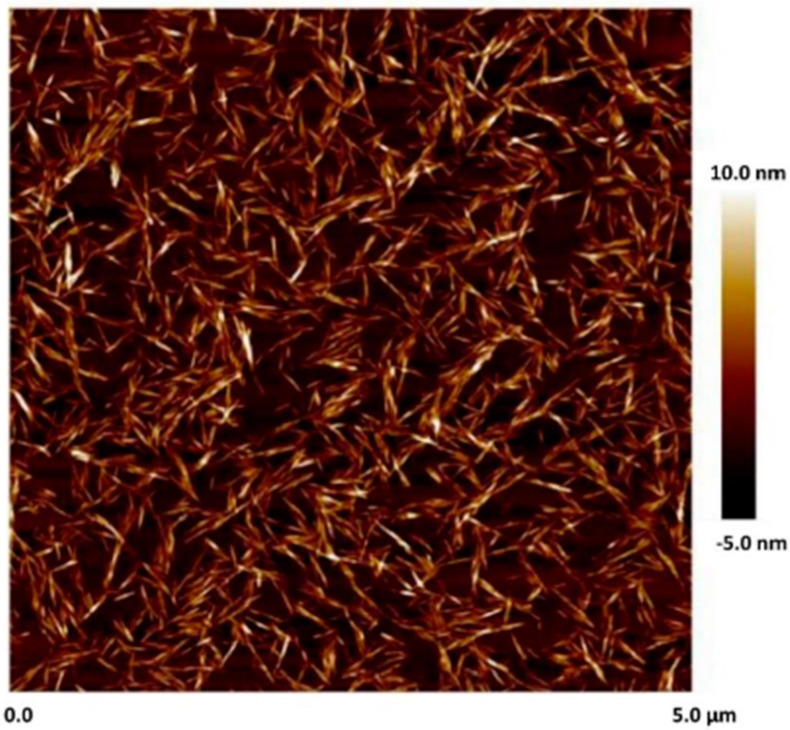
AFM image of NCC [36].

CelluForce Inc., Windsor, ON, Canada, supplied the NCC (trade name NCC NCV100-NASD90) used in this work. The NCC was manufactured using sulfuric acid hydrolysis of wood pulp. The atomic force microscopy (AFM) image of cellulose nanocrystals is shown in Figure 1. The nanocrystals are rod-shaped particles with the mean length and width of 76 nm and 3–4 nm, respectively [36].

### 2.2. Preparation of Polymer Solutions

Solutions of polymer were prepared in batches of approximately 1 kg at room temperature (≅22.5 °C) by adding a known amount of polymer to a known amount of de-ionized water. Gentle mixing of the solution was maintained during the addition of polymer using a turbine homogenizer (Gifford-Wood, model 1L, NOV process and flow technologies, Dayton, OH, USA). The solution was finally homogenized in the homogenizer at a fixed high speed. The speed of the homogenizer was controlled by varying the voltage of the homogenizer using a variac. The variac setting was fixed at about 30% of the full scale. The solution was sheared for at least 30 min at a fixed speed. In some cases (depending on polymer and polymer concentration), the duration of mixing was longer than 30 min. Each polymer solution at different concentrations was freshly prepared. The solution was left unstirred at room temperature for at least three hours before any measurements were made to eliminate any air entrapped during mixing and to cool down the sample to room temperature.

### 2.3. Preparation of Mixtures of Cellulose Nanocrystals and Polymer Solutions

The mixtures of NCC and polymer solutions were prepared in batches of approximately 1 kg at room temperature (≅22.5 °C) by slowly dispersing a known amount of NCC into a known amount of polymer solution. The mixture was homogenized at the same fixed speed (variac setting 30% of full scale) as that used in the preparation of the polymer solution for a duration of at least 30 min. In some cases, the duration of mixing was extended to ensure thorough mixing of the components. To increase the NCC concentration of the mixture, the required amount of more NCC was added slowly to the known amount of an existing lower NCC concentration mixture and the mixture was homogenized at a fixed speed (variac setting 30% of full scale) for at least 30 min. The duration of mixing was extended in some cases to ensure thorough mixing of the components. As in the case of polymer solution alone, the NCC–polymer mixture was left unstirred at room temperature for at least three hours before any measurements were made to eliminate any air entrapped during mixing and to cool down the sample to room temperature.

### 2.4. Measurements

Two Fann co-axial cylinder viscometers with different torsion spring constants and a Haake co-axial cylinder viscometer with three different bobs (inner cylinders) were used to measure the rheological properties. It was necessary to use different devices (Fann and Haake) and bobs to cover a broad range of viscosities exhibited by the fluids. Table 2 gives the relevant dimensions of the viscometers used in this work. The rotational speed varied from 0.9 to 600 rpm in the Fann viscometer and from 0.01 to 512 rpm in the Haake viscometer. The viscometers were calibrated using viscosity standards of known viscosities. All the viscosity measurements were carried out at room temperature (≈22.5 °C).

The key difference between the Fann and Haake viscometers is that different components rotate in the two viscometers. They are both co-axial cylinder-type viscometers. In the Fann viscometer, the inner cylinder (smaller diameter) is stationary, and the outer cylinder (larger diameter) rotates. In this case, the torque generated on the inner cylinder is measured and related to fluid viscosity. In the Haake viscometer, the inner cylinder (smaller diameter) is rotated, and the outer cylinder (larger diameter) is kept stationary. The torque generated on the inner cylinder is measured and related to fluid viscosity. The torque ranges of the two instruments were different so that they could together cover a broad range of viscosities

For non-homogeneous fluids, such as concentrated suspensions, the gap width of the viscometer can affect the measured viscosities as heterogeneous suspensions are prone to slip effects. However, the present fluids were homogeneous with no slip effects. The viscosities measured by the two different instruments (Fann and Haake) and different gap widths agreed with each other.

## 3. Experimental Results and Discussion

### 3.1. Rheology of CMC Polymer Solutions and NCC–CMC Mixtures

**Figure 2:**
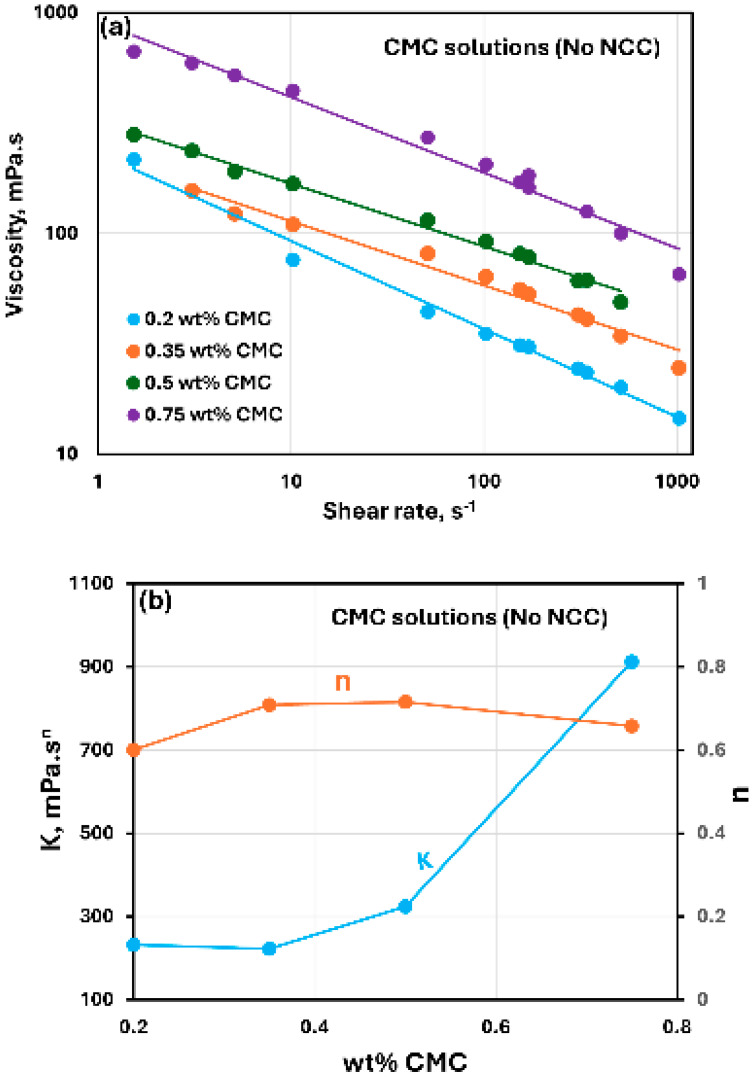
Rheological behavior of CMC polymer solutions without any NCC addition. (**a**) Viscosity versus shear rate, (**b**) Power-law parameters K and n.

Figure 2 shows the rheological data for CMC polymer solutions without any NCC addition. The polymer solutions are non-Newtonian shear-thinning, that is, the viscosity decreases with the increase in the shear rate. The shear-thinning flow behavior of CMC polymer solutions observed here is consistent with the published studies on the rheology of CMC solutions [48,49,50,51,52]. The shear-thinning behavior of polymer solutions is due to the stretching and alignment of polymer molecules with shear in the direction of flow. Viscosity versus shear rate data for polymer solutions follow a linear relationship on a log-log plot, see Figure 2a, indicating power-law behavior. The power-law model is given as:

(1) $$\tau =K{\dot{\gamma }}^{n}$$

(2) $$\mu =\frac{\tau }{\dot{\gamma }}=K{\dot{\gamma }}^{n-1}$$

where τ is shear stress, $\dot{\gamma }$ is shear rate, K is consistency index, and n is flow behavior index. Figure 2b shows the variations in power-law constants (K and n) of polymer solutions with CMC concentration. The consistency index K increases with the increase in CMC concentration, especially at high concentrations. The flow behavior index n is less than one as expected for a shear-thinning fluid. It varies only marginally with the increase in CMC concentration.

**Figure 3:**
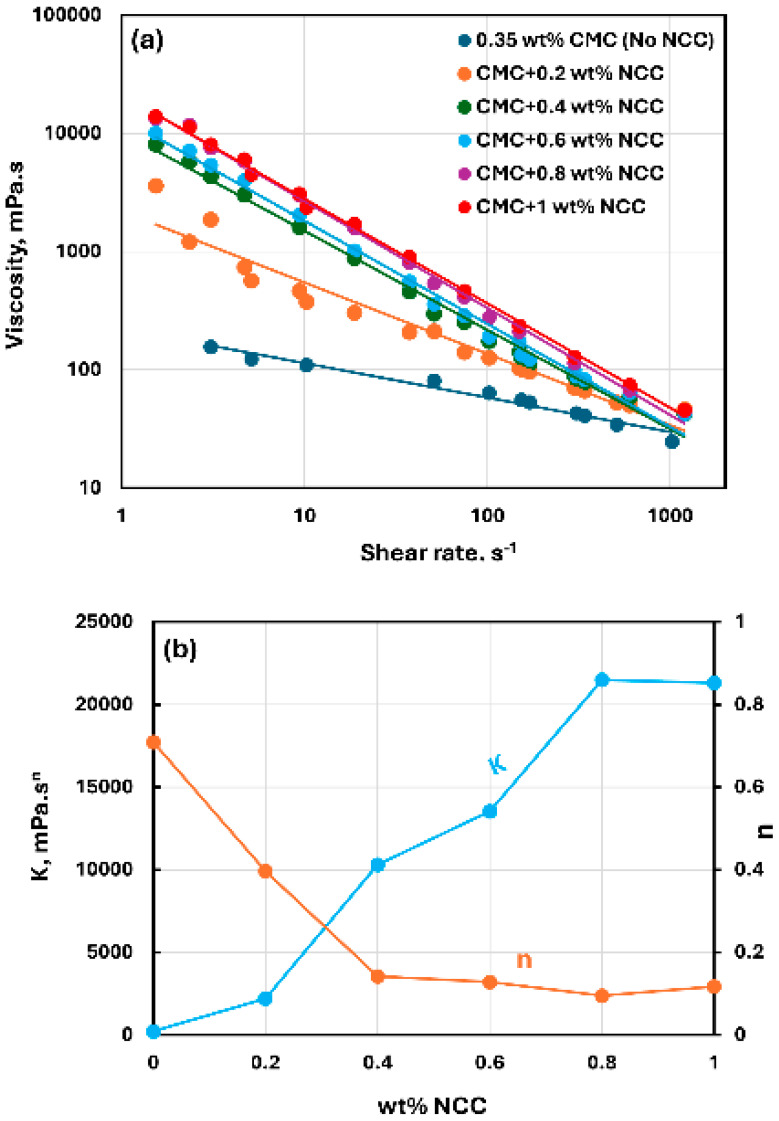
Rheological behavior of NCC–CMC mixtures at a fixed CMC concentration of 0.35 wt%. (**a**) Viscosity versus shear rate, (**b**) Power-law parameters K and n.

**Figure 4:**
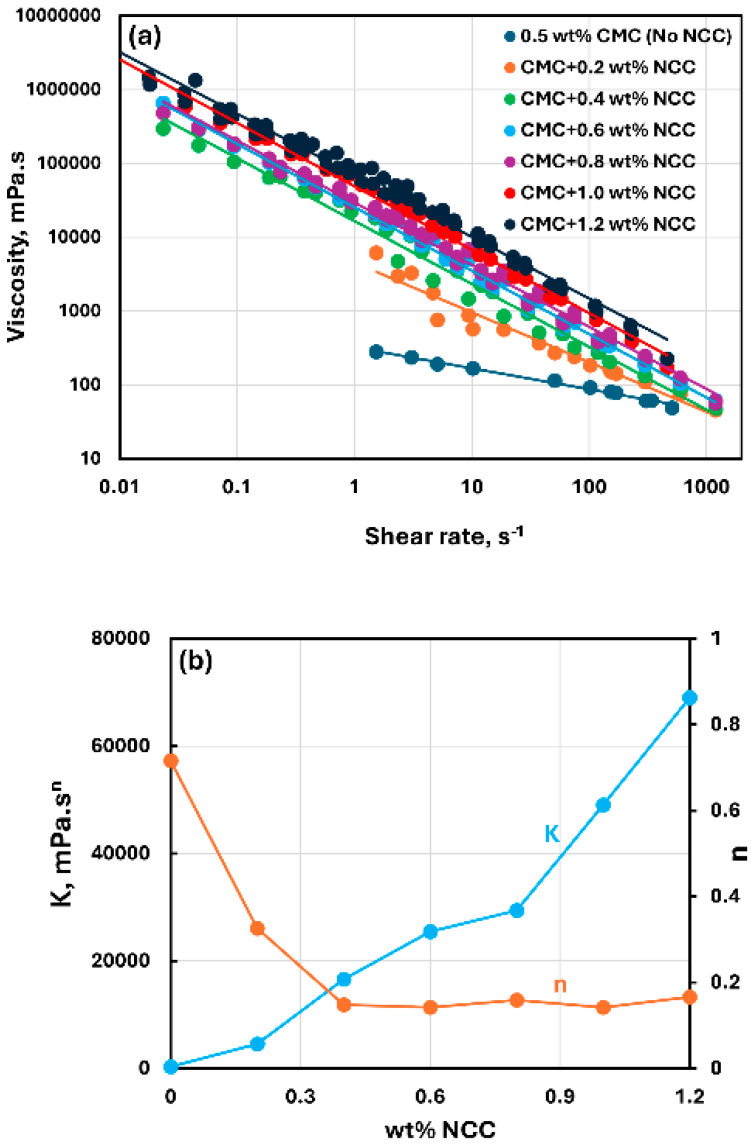
Rheological behavior of NCC–CMC mixtures at a fixed CMC concentration of 0.5 wt%. (**a**) Viscosity versus shear rate, (**b**) Power-law parameters K and n.

**Figure 5:**
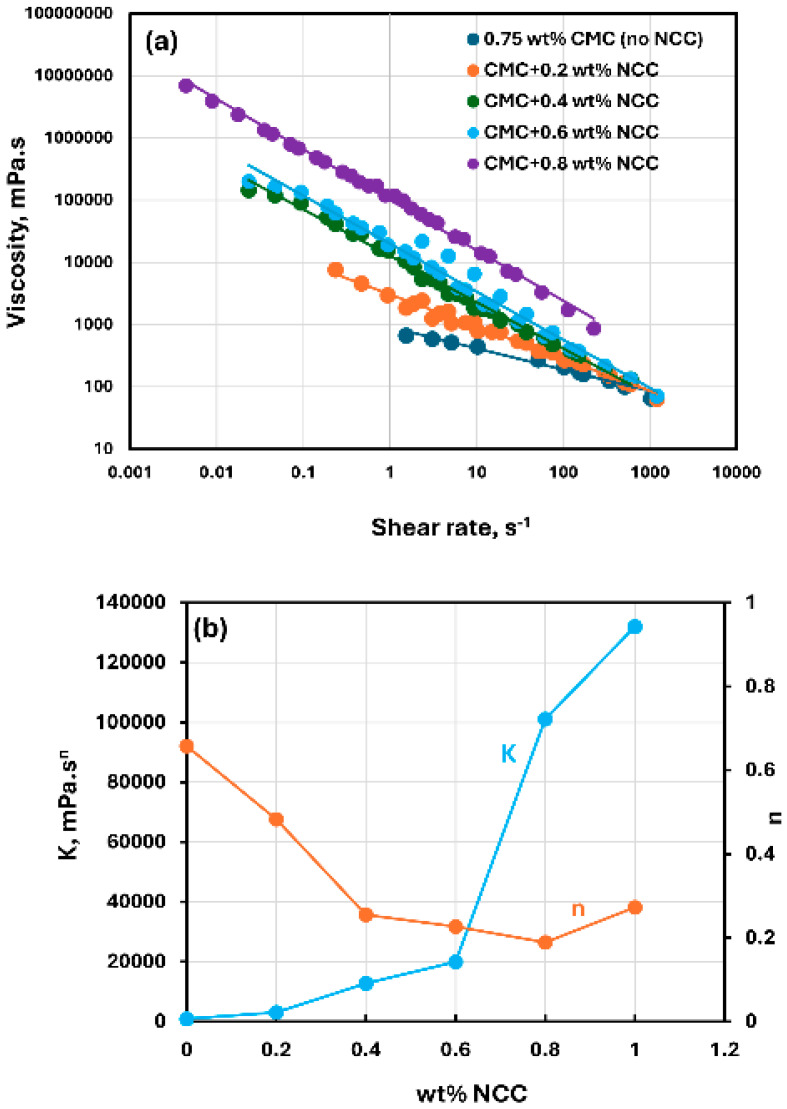
Rheological behavior of NCC–CMC mixtures at a fixed CMC concentration of 0.75 wt%. (**a**) Viscosity versus shear rate, (**b**) Power-law parameters K and n.

Figure 3, Figure 4 and Figure 5 show the rheological behavior of mixtures of NCC and CMC polymer solutions. The NCC concentration varies from 0 to as high as 1.2 wt% in increments of 0.2 wt%. The flow curves (viscosity versus shear rate plots) for NCC–CMC mixtures can be described well by the power-law model, Equation (2). The addition of NCC to CMC solution has a strong influence on the consistency and flow behavior of the system as reflected in the power law parameters: K and n. At a given CMC concentration, the consistency index K increases sharply whereas the flow behavior index n decreases sharply with the increase in NCC concentration. Thus, the addition of cellulose nanocrystals to CMC polymer solution makes the system more viscous and shear-thinning.

**Figure 6:**
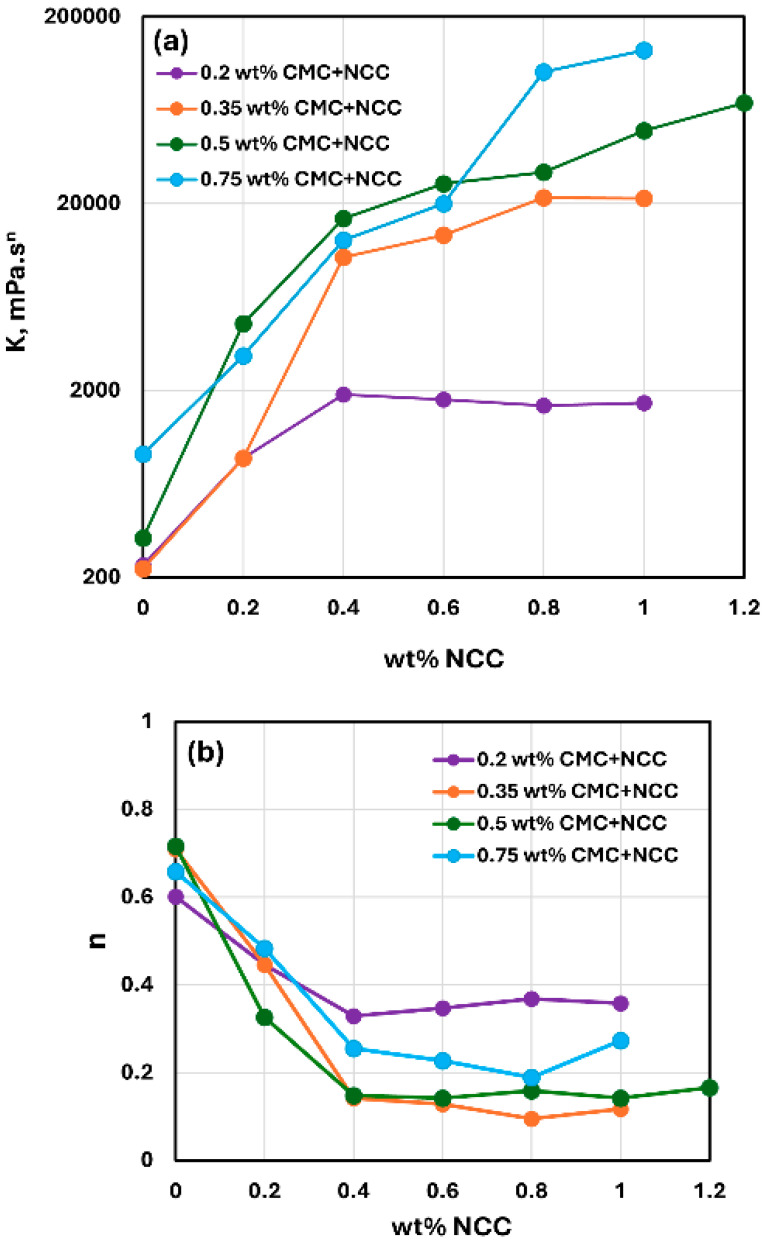
Comparison of consistency index K and flow behavior index n of NCC–CMC mixtures at different CMC concentrations. (**a**) Consistency index K, (**b**) Flow behavior index n.

Figure 6 compares the consistency index K and flow behavior index n values for NCC–CMC mixtures with different concentrations of CMC. With the increase in CMC polymer concentration, the consistency index K generally increases at any given NCC concentration, especially at high NCC concentrations. No clear trend is observed in the flow behavior index n with the increase in CMC concentration.

**Figure 7:**
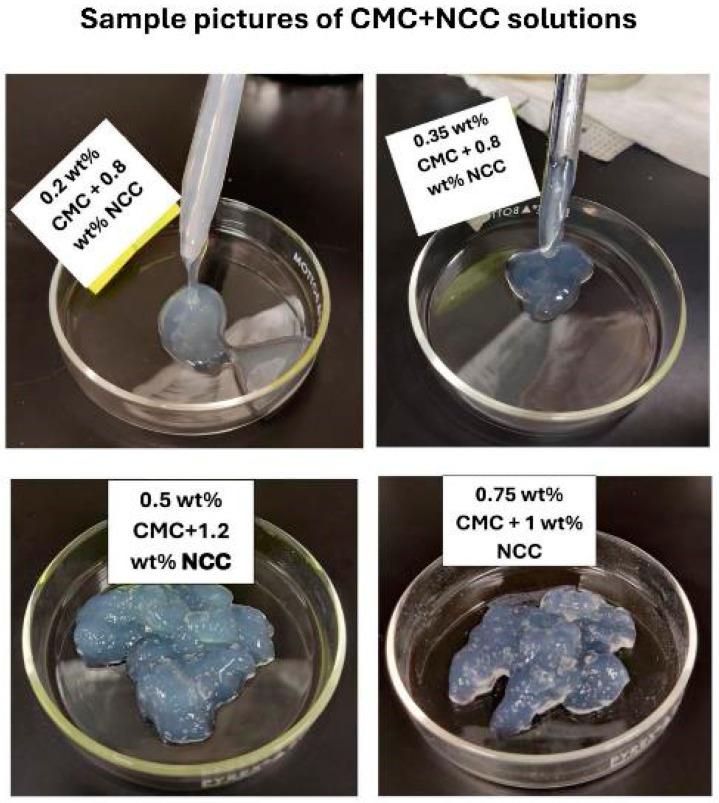
Images of samples of NCC–CMC mixtures.

Figure 7 shows the images of the samples of NCC–CMC mixtures. The mixture is fluidic at low CMC and NCC concentrations. The mixture appears as a gel at high CMC and NCC concentrations.

### 3.2. Rheology of Guar Gum Solutions and NCC–Guar Gum Mixtures

**Figure 8:**
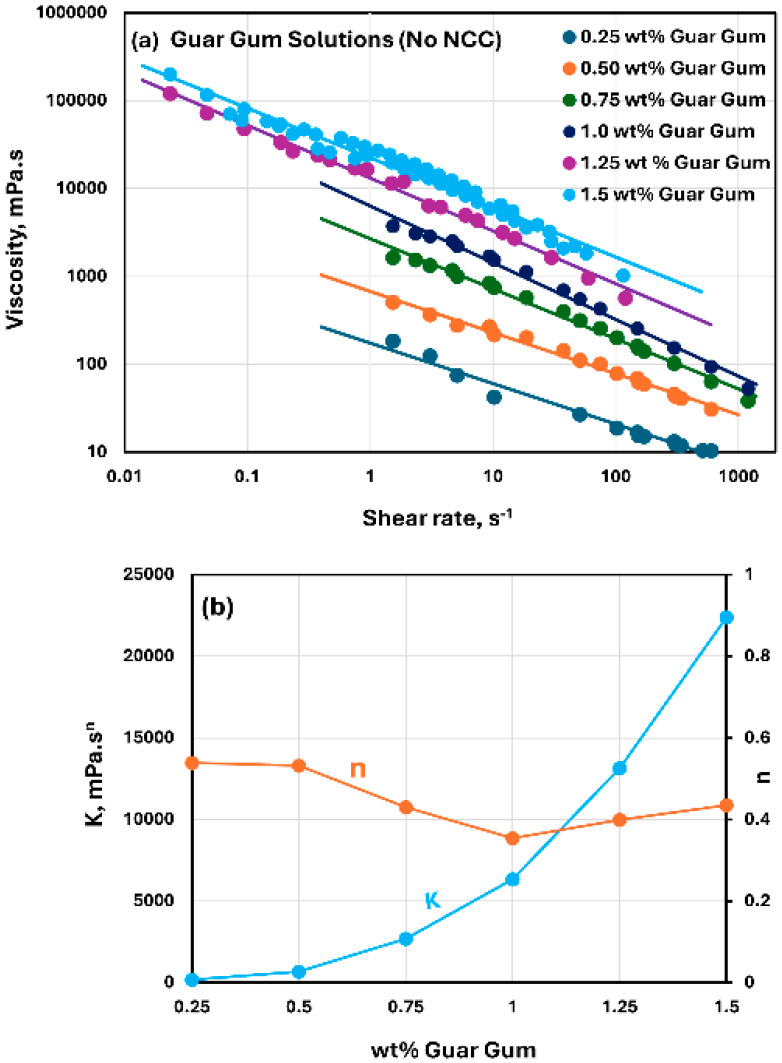
Rheological behavior of guar gum solutions without any NCC addition. (**a**) Viscosity versus shear rate, (**b**) Power-law parameters K and n.

The rheological behavior of guar gum solutions without any NCC addition is shown in Figure 8. The guar gum solutions are highly shear-thinning, and they follow the power-law model, Equation (2). The consistency index K increases with the increase in guar gum concentration. The flow behavior index n is in the range of 0.35–0.54 over the guar gum concentration investigated. A relatively low value of n is indicative of a high degree of shear-thinning in guar gum solutions. The shear-thinning behavior of guar gum solutions observed here agrees with the available literature on the rheology of guar gum solutions [53,54,55].

**Figure 9:**
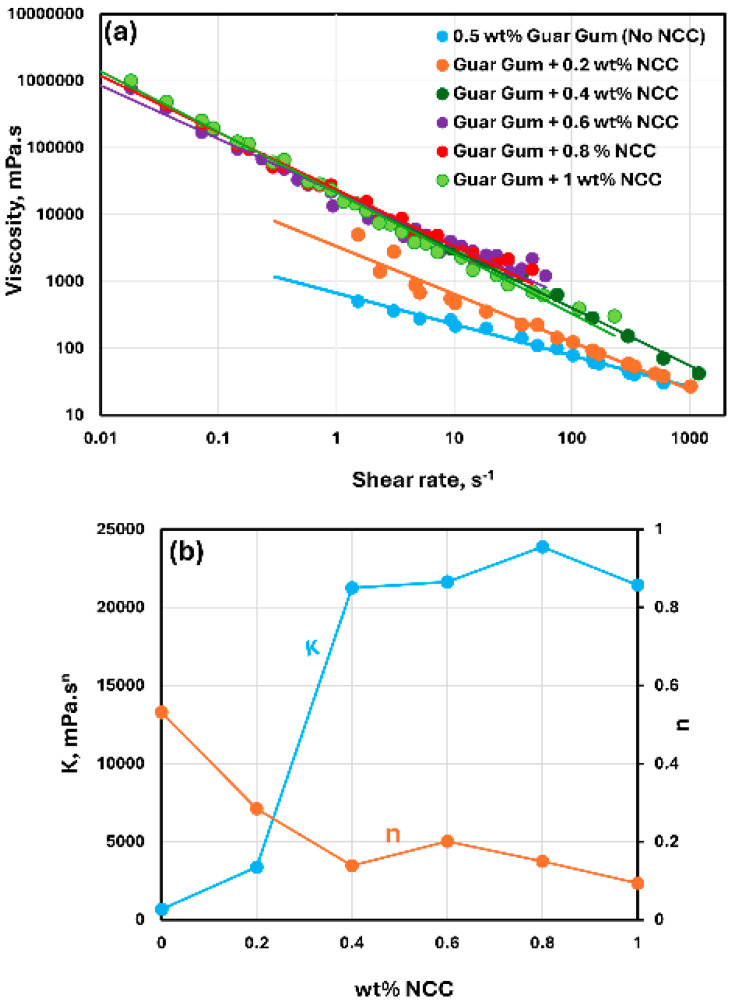
Rheological behavior of NCC–guar gum mixtures at a fixed guar gum concentration of 0.5 wt%. (**a**) Viscosity versus shear rate, (**b**) Power-law parameters K and n.

**Figure 10:**
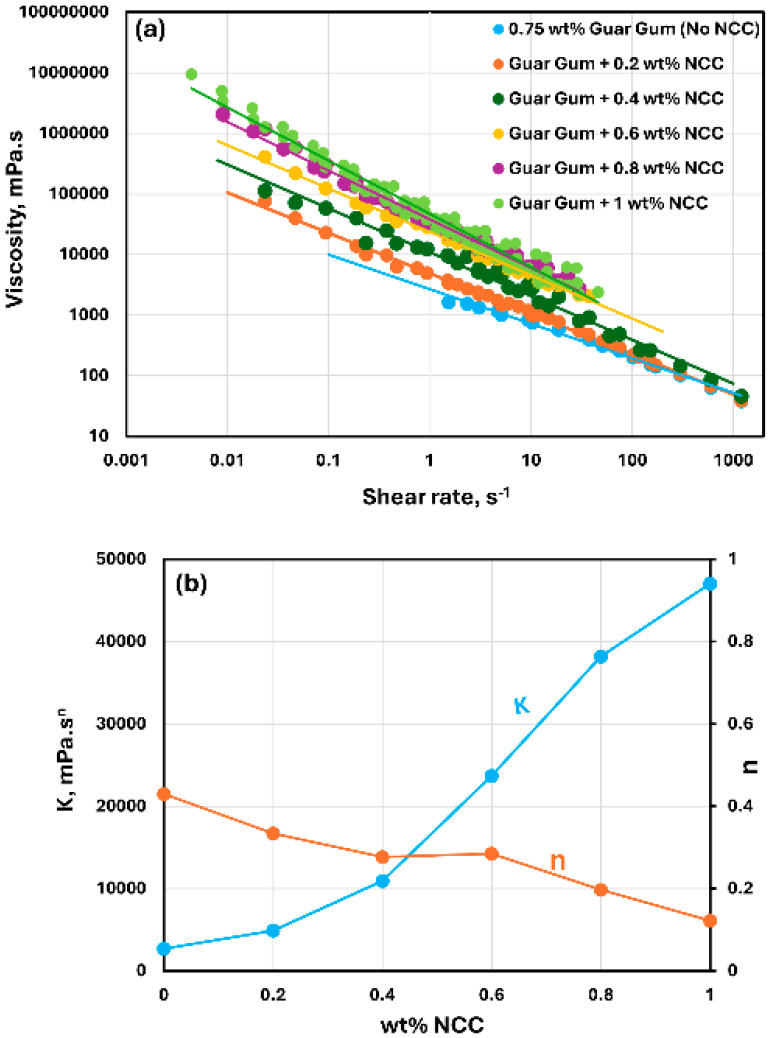
Rheological behavior of NCC–guar gum mixtures at a fixed guar gum concentration of 0.75 wt%. (**a**) Viscosity versus shear rate, (**b**) Power-law parameters K and n.

**Figure 11:**
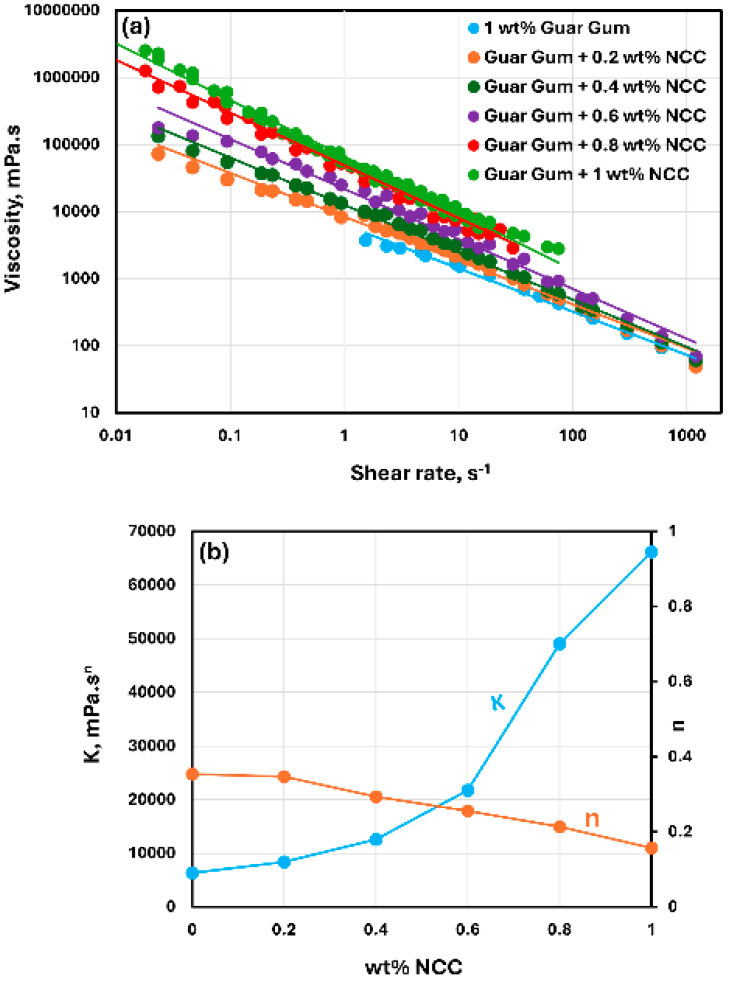
Rheological behavior of NCC–guar gum mixtures at a fixed guar gum concentration of 1 wt%. (**a**) Viscosity versus shear rate, (**b**) Power-law parameters K and n.

The rheological behavior of mixtures of NCC and guar gum solution are shown in Figure 9, Figure 10 and Figure 11. The NCC concentration varies from 0 to 1.0 wt% in increments of 0.2 wt%. The mixtures of NCC and guar gum solution are highly shear-thinning. The viscosity versus shear rate data for NCC–guar gum mixtures can be described well by the power-law model, Equation (2). The consistency index K increases sharply and the flow behavior index n decreases with the increase in NCC concentration at any given guar gum concentration. Thus, the mixture of NCC and guar gum solution becomes more viscous and shear-thinning with the addition of cellulose nanocrystals to the guar gum solution.

**Figure 12:**
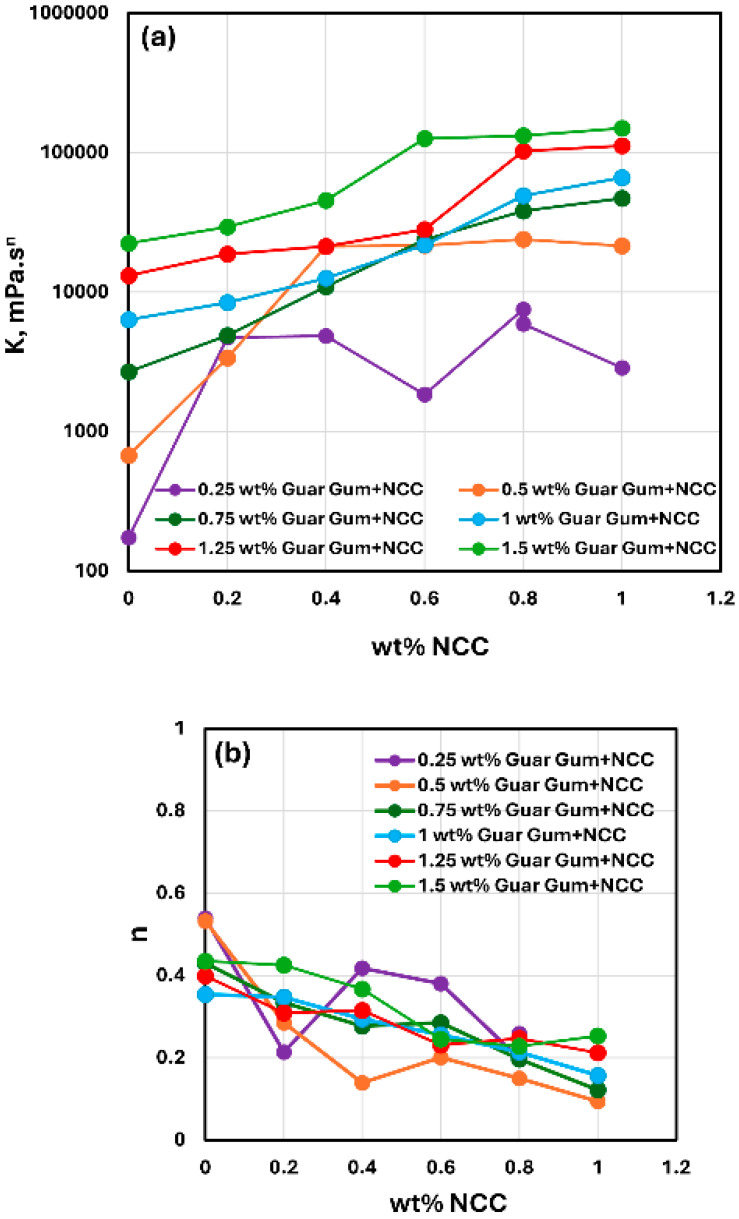
Comparison of consistency index K and flow behavior index n of NCC–guar gum mixtures at different guar gum concentrations. (**a**) Consistency index K, (**b**) Flow behavior index n.

The consistency index K and flow behavior index n values for NCC–guar gum mixtures with different concentrations of guar gum are compared in Figure 12. With the increase in guar gum concentration, the consistency index K generally increases at any given NCC concentration. No clear trend is observed in the flow behavior index n with the increase in guar gum concentration. However, the flow behavior index generally decreases with the increase in NCC concentration at any given guar gum concentration.

**Figure 13:**
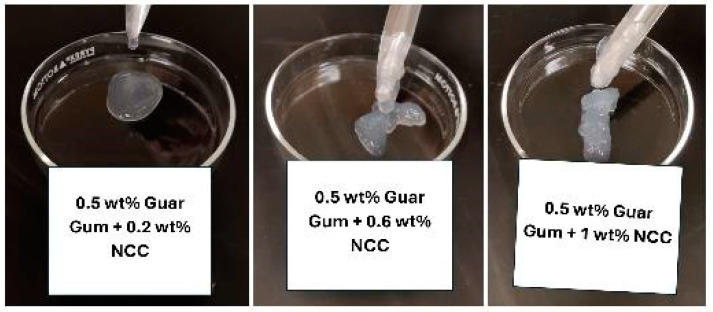
Images of samples of NCC–guar gum mixtures.

The images of the samples of NCC–guar gum mixtures with increasing NCC concentration but fixed guar gum concentration of 0.5 wt% are shown in Figure 13. The mixture is fluidic at low CMC and NCC concentrations. The mixture is fluidic at low NCC concentration but becomes a gel at high concentration keeping the guar gum concentration constant.

### 3.3. Rheology of WSR-303 Solutions and NCC–WSR Mixtures

**Figure 14:**
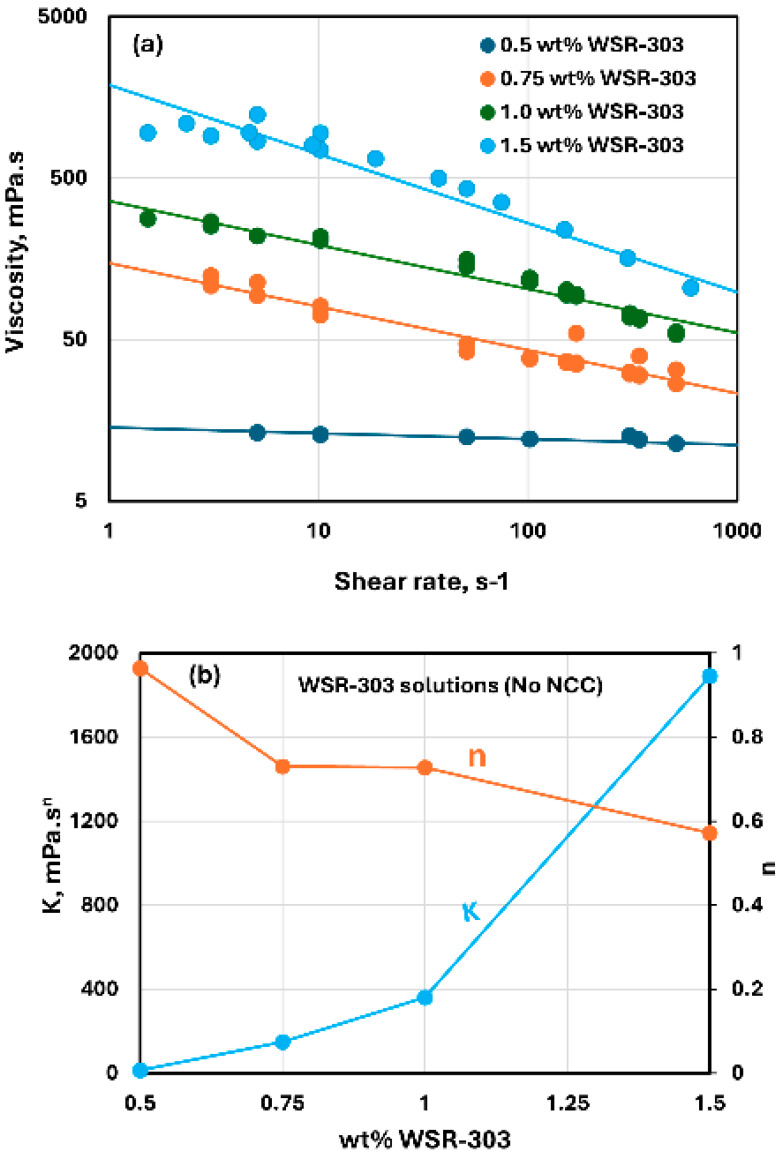
Rheological behavior of WSR-303 solutions without any NCC addition. (**a**) Viscosity versus shear rate, (**b**) Power-law parameters K and n.

The flow curves of WSR-303 solutions without any NCC addition are shown in Figure 14. The WSR-303 solutions are shear-thinning, and they follow the power-law model, Equation (2). The consistency index K increases and the flow behavior index n decreases with the increase in WSR-303 concentration. This observation is consistent with the literature on flow behavior of WSR-303 solutions [56]. The flow behavior index n of the WSR-303 solutions is in the range of 0.57–0.96 over the WSR-303 concentration investigated. Note that WSR-303 solutions are less shear-thinning as compared with CMC and guar gum solutions. For example, the flow behavior index n of the guar gum is significantly lower in the range of 0.35–0.54 over the guar gum concentration investigated.

**Figure 15:**
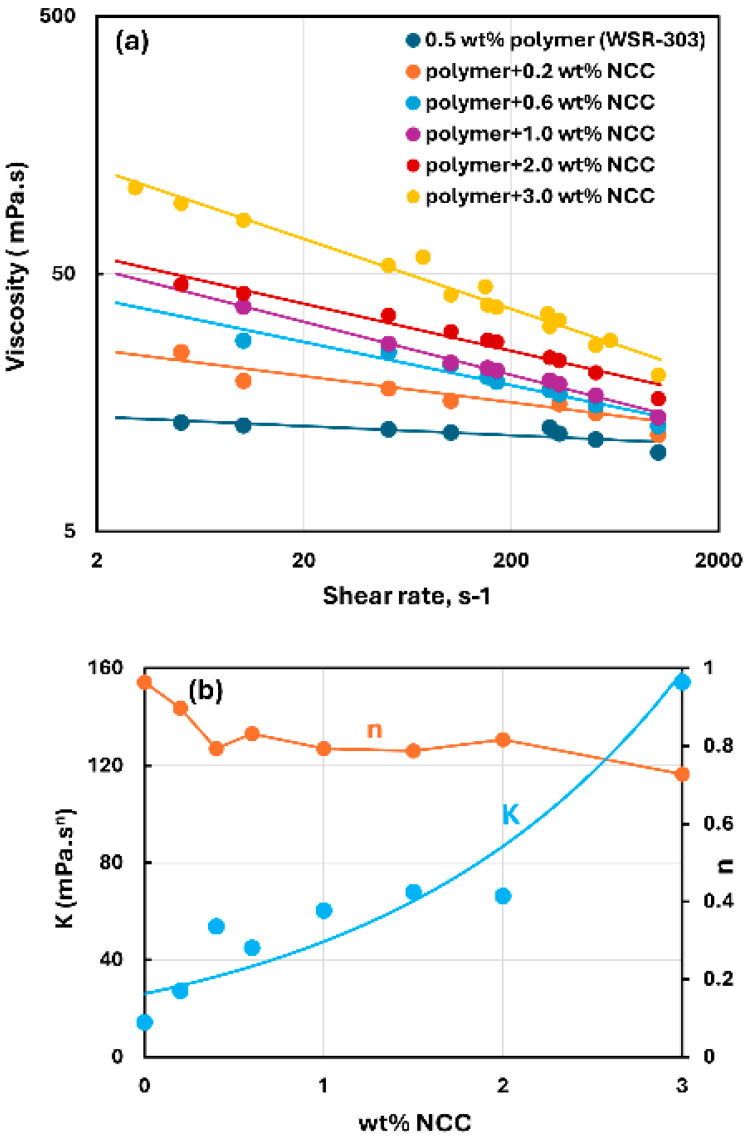
Rheological behavior of NCC–WSR-303 mixtures at a fixed WSR-303 concentration of 0.5 wt%. (**a**) Viscosity versus shear rate, (**b**) Power-law parameters K and n.

**Figure 16:**
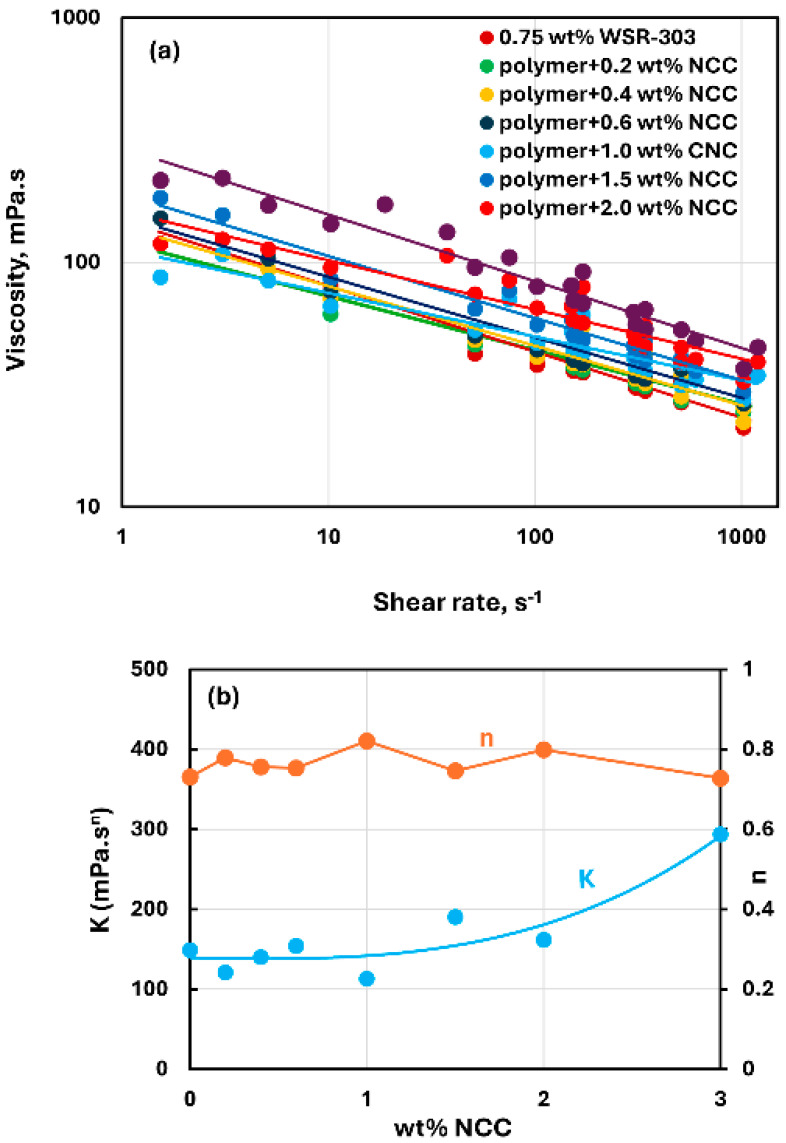
Rheological behavior of NCC–WSR-303 mixtures at a fixed WSR-303 concentration of 0.75 wt%. (**a**) Viscosity versus shear rate, (**b**) Power-law parameters K and n.

**Figure 17:**
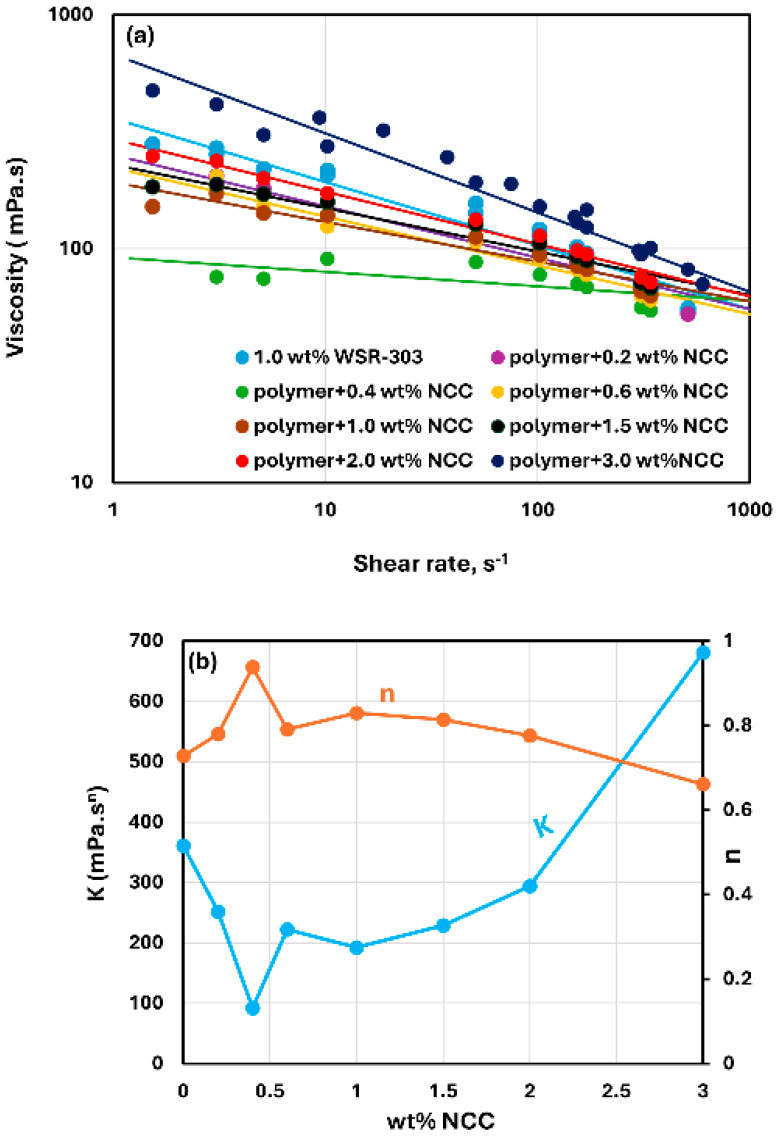
Rheological behavior of NCC–WSR-303 mixtures at a fixed WSR-303 concentration of 1 wt%. (**a**) Viscosity versus shear rate, (**b**) Power-law parameters K and n.

Figure 15, Figure 16 and Figure 17 show the rheological behavior of mixtures of NCC and WSR-303 polymer solutions. The NCC concentration varies from 0 to 3 wt%. The flow curves (viscosity versus shear rate plots) for NCC–WSR-303 mixtures can be described satisfactorily by the power-law model, Equation (2). For polymer (WSR-303) concentrations at 0.5 wt% (Figure 15) and 0.75 wt% (Figure 16), the addition of NCC to WSR-303 solution causes an increase in the consistency index and a decrease in the flow behavior index of the system. However, the increase in K and a decrease in n with the increase in NCC concentration are only modest. At a WSR-303 concentration of 1 wt% (Figure 17), the consistency index K and flow behavior index n exhibit unusual behavior. The consistency index (K) decreases initially with the increase in NCC concentration, reaches a minimum value and rises with a further increase in NCC concentration. The flow behavior index (n) increases initially to a maximum value before decreasing with the increase in NCC concentration.

**Figure 18:**
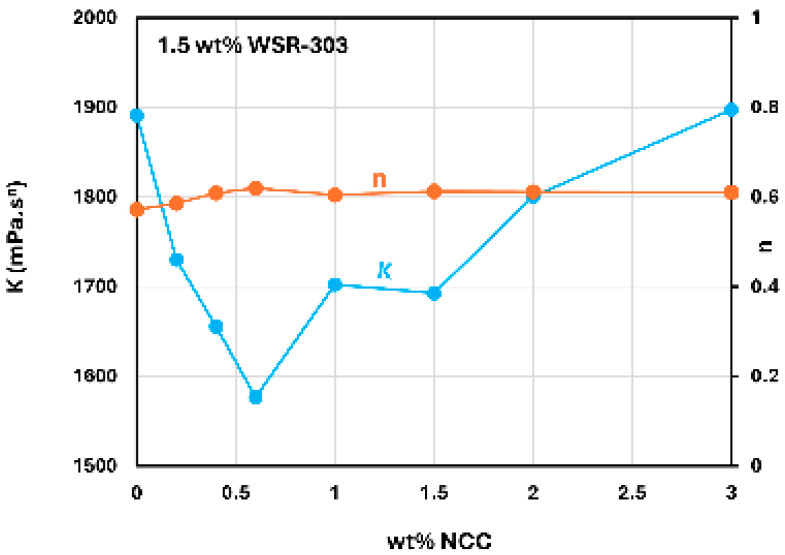
Consistency index (K) and flow behavior index (n) of NCC–WSR-303 mixtures at a fixed WSR-303 concentration of 1.5 wt%.

Figure 18 shows the consistency index (K) and flow behavior index (n) of NCC–WSR-303 mixtures at a WSR-303 concentration of 1.5 wt%. With the increase in NCC concentration, the consistency index (K) goes through a sharp minimum whereas the flow behavior index (n) remains nearly constant around 0.6.

**Figure 19:**
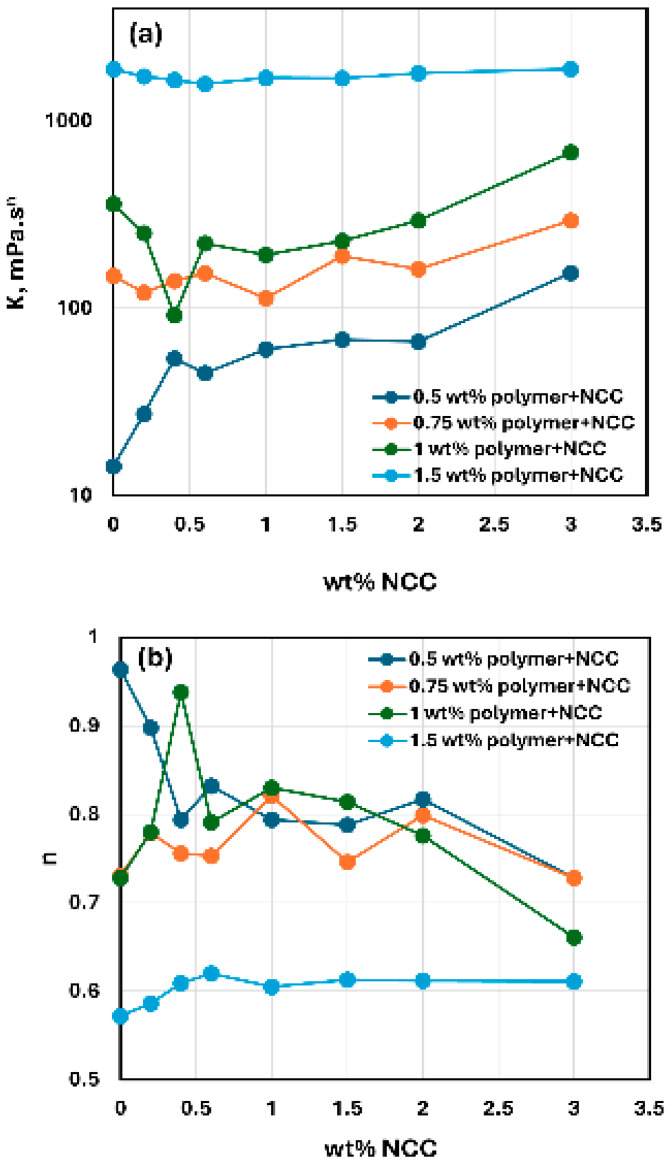
Comparison of consistency index K and flow behavior index n of NCC–WSR-303 mixtures at different concentrations of WSR-303. (**a**) Consistency index K, (**b**) Flow behavior index n.

Figure 19 compares the consistency index K and flow behavior index n values for NCC–WSR-303 mixtures with different concentrations of WSR-303. At NCC concentrations higher than 1 wt%, the consistency index K increases with the increase in NCC concentration at any given WSR-303 concentration. At NCC concentrations lower than 1 wt%, the consistency index K exhibits a minimum for mixtures with WSR-303 concentrations 1 wt% and 1.5 wt%. The flow behavior index n of NCC–WSR-303 mixtures is similar when WSR-303 concentration is less than 1 wt%. A sharp drop in n occurs when the WSR-303 concentration increases from 1 wt% to 1.5 wt%, indicating that NCC–WSR-303 mixtures are highly shear-thinning at a high WSR-303 concentration of 1.5 wt% over the full range of NCC concentration investigated.

### 3.4. Rheology of Xanthan Gum Solutions and NCC–Xanthan Gum Mixtures

**Figure 20:**
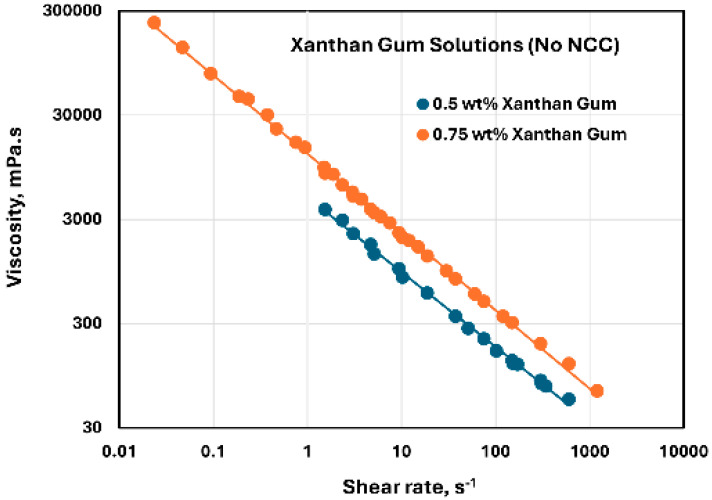
Rheological behavior of xanthan gum solutions without any NCC addition.

The rheological behavior of xanthan gum solutions without any NCC addition is shown in Figure 20. The data were obtained at two xanthan gum concentrations of 0.5 and 0.75 wt%. As shown in the figure, the xanthan gum solutions are highly shear-thinning, and they follow the power-law model, Equation (2). The consistency index K increases substantially with the increase in xanthan gum concentration from 0.5 to 0.75 wt%. The value of K is 4888.3 mPa.s^n^ at xanthan gum concentration of 0.5 wt% and 12,798 mPa.s^n^ at xanthan gum concentration of 0.75 wt%. The flow behavior index n decreases from 0.282 at xanthan gum concentration of 0.5 wt% to 0.249 at xanthan gum concentration of 0.75 wt%. The rheological behavior of xanthan gum solutions observed here is consistent with the available literature on the rheology of xanthan gum solutions [57,58].

**Figure 21:**
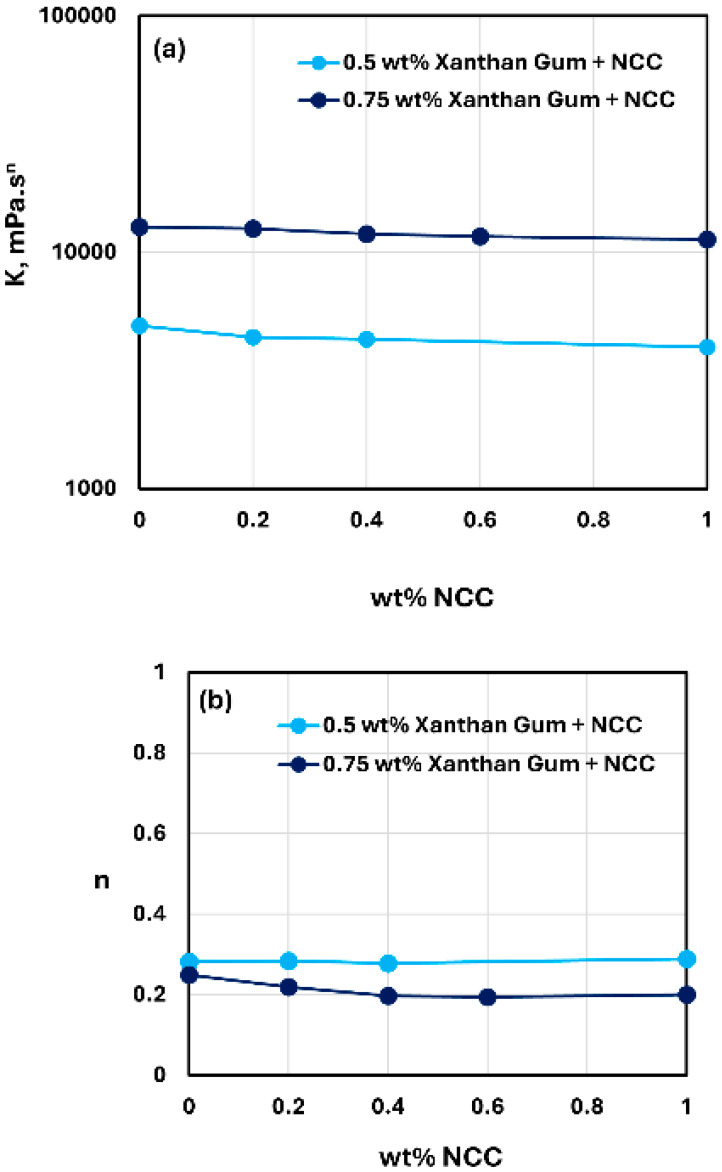
Comparison of consistency index K and flow behavior index n of NCC–xanthan gum mixtures at different concentrations of xanthan gum. (**a**) Consistency index K, (**b**) Flow behavior index n.

The rheological behavior of mixtures of NCC and xanthan gum solutions was studied over the NCC concentration range of 0 to 1 wt%. The viscosity versus shear rate data obtained for NCC–xanthan gum mixtures was fitted satisfactorily by the power-law model, Equation (2). The consistency index K and flow behavior index n values for NCC–xanthan gum mixtures with different concentrations of xanthan gum are compared in Figure 21. At any given xanthan gum concentration, the consistency index K and flow behavior index n are almost constant with the increase in NCC concentration. Thus, NCC addition to xanthan gum solutions has negligible effect on the rheology of NCC–xanthan gum mixtures.

### 3.5. Rheology of Praestol 2505 Solutions and NCC–Praestol 2505 Mixtures

**Figure 22:**
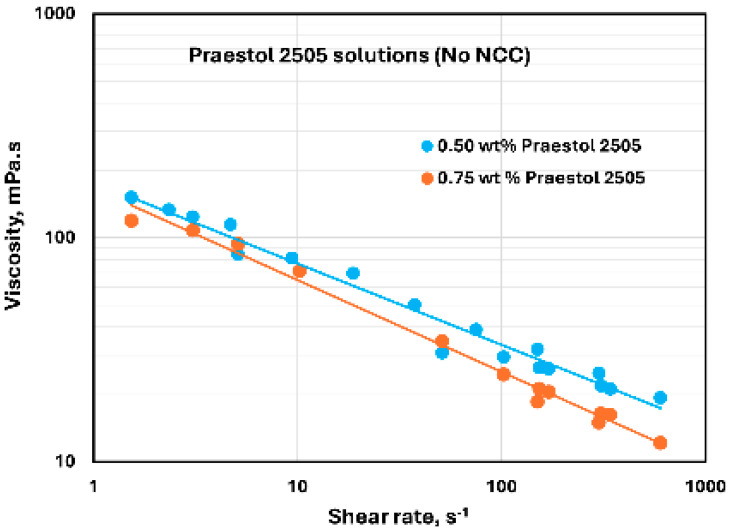
Rheological behavior of Praestol 2505 polymer solutions without any NCC addition.

The flow curves of Praestol 2505 polymer (anionic polyacrylamide) solutions without any NCC addition are shown in Figure 22 at two different polymer concentrations of 0.5 and 0.75 wt%. The Praestol 2505 polymer solutions are shear-thinning, and they follow the power-law model, Equation (2). The consistency index K values are 176.73 and 166.45 mPa.s^n^ at 0.5 and 0.75 wt% polymer concentrations, respectively. The flow behavior index n values of the Praestol 2505 polymer solutions are 0.637 and 0.59 at 0.5 and 0.75 wt% polymer concentrations, respectively. Note that anionic polyacrylamide solutions are known to behave as shear-thinning fluids as observed here [59].

**Figure 23:**
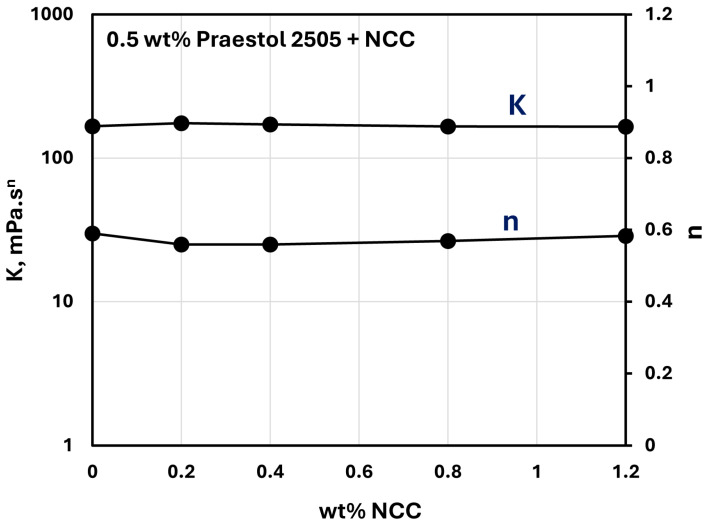
Variations in consistency index K and flow behavior index n of NCC–Praestol 2505 mixtures with NCC concentration at Praestol 2505 concentration of 0.5 wt%.

**Figure 24:**
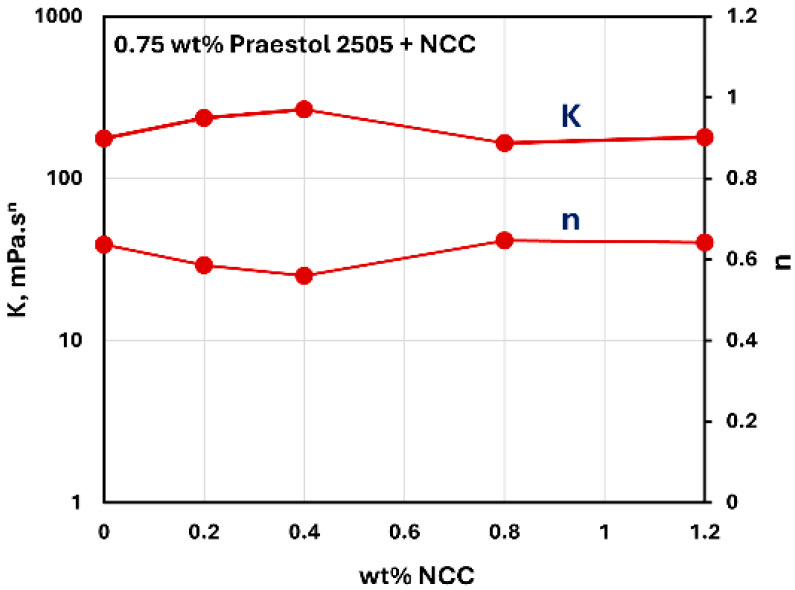
Variations in consistency index K and flow behavior index n of NCC–Praestol 2505 mixtures with NCC concentration at Praestol 2505 concentration of 0.75 wt%.

Figure 23 and Figure 24 show the influence of the addition of NCC on the consistency index K and flow behavior index n of NCC–Praestol 2505 mixtures with NCC concentration at two different concentrations of Praestol 2505. The consistency index K and flow behavior index n are nearly constant with the increase in NCC concentration. Thus, the addition of cellulose nanocrystals to Praestol 2505 polymer solutions is negligible.

### 3.6. Rheology of JR-400 Solutions and NCC–JR-400 Mixtures

**Figure 25:**
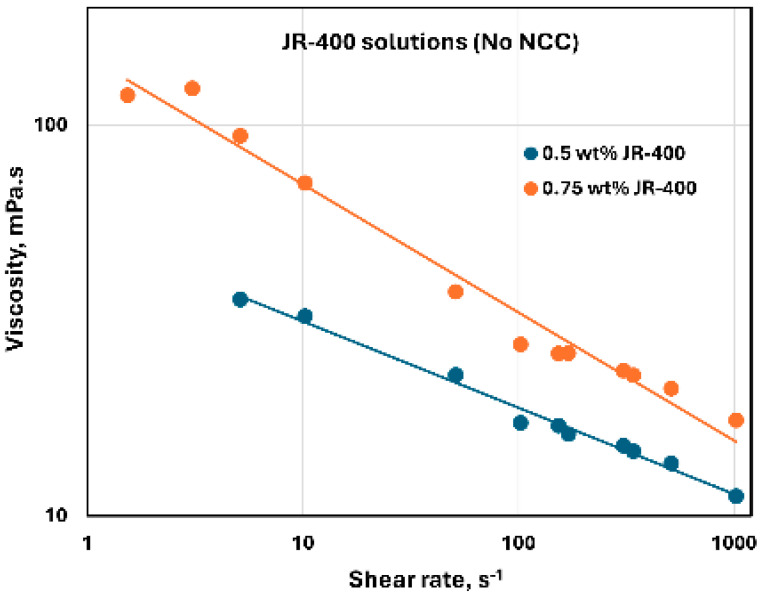
Rheological behavior of JR-400 polymer solutions without any NCC addition.

The rheological behavior of JR-400 polymer solutions without any NCC addition is shown in Figure 25. The data were obtained at two JR-400 concentrations of 0.5 and 0.75 wt%. The JR-400 polymer solutions are shear-thinning, and they follow the power-law model, Equation (2). The consistency index K increases substantially with the increase in JR-400 concentration from 0.5 to 0.75 wt%. The value of K is 52.32 mPa.s^n^ at JR-400 concentration of 0.5 wt% and 150.08 mPa.s^n^ at JR-400 concentration of 0.75 wt%. The flow behavior index n decreases from 0.779 at JR-400 concentration of 0.5 wt% to 0.672 at JR-400 concentration of 0.75 wt%. The rheological behavior of JR-400 polymer solutions observed here agrees with the literature study [60].

**Figure 26:**
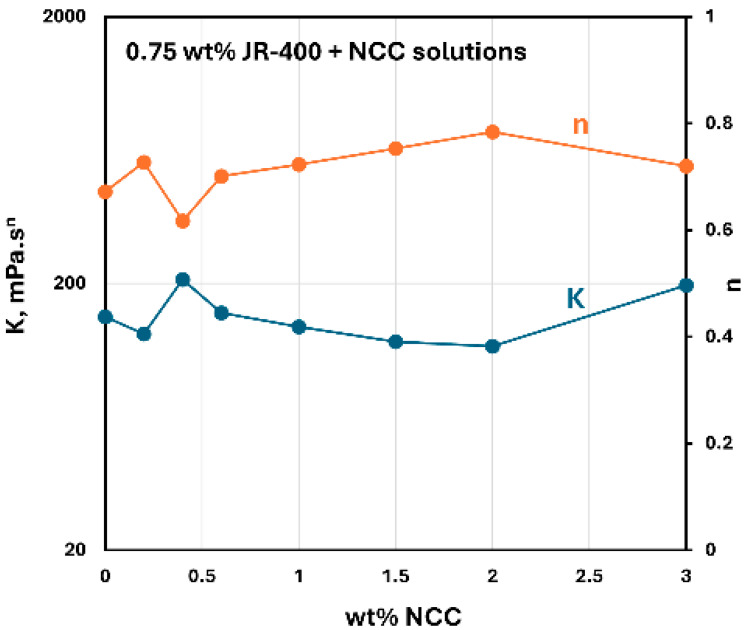
Variations in consistency index K and flow behavior index n of NCC–JR-400 mixtures with NCC concentration at JR-400 concentration of 0.75 wt%.

**Figure 27:**
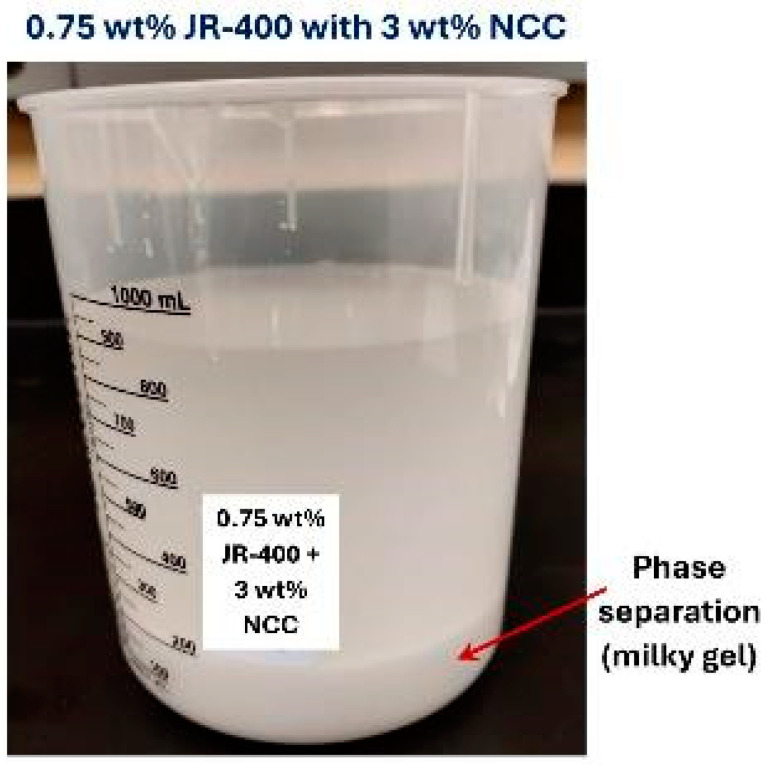
Phase separation in JR-400–NCC mixture. The JR-400 concentration is 0.75 wt% and NCC concentration is 3 wt%.

The influence of the addition of NCC to JR-400 polymer solution is shown in Figure 26. The power-law constants K and n for NCC–JR-400 mixtures are plotted as a function of NCC concentration, keeping the JR-400 polymer concentration constant at 0.75 wt%. As can be seen, the consistency index K and flow behavior index n remain nearly constant with the NCC addition. Thus, the addition of cellulose nanocrystals to JR-400 polymer solution has a negligible effect on the rheological properties of the NCC–JR-400 mixture. Interestingly, phase separation (see Figure 27) occurred in NCC–JR-400 mixture when the NCC concentration of the mixture was increased to 3 wt%. A thick layer of milky gel (concentrated suspension of cellulose nanocrystals in polymeric aqueous phase) was observed at the bottom of the container. This observation is not unexpected as the NCC and JR-400 polymer are oppositely charged, and they likely form aggregates and precipitate out.

## 4. Discussion

**Figure 28:**
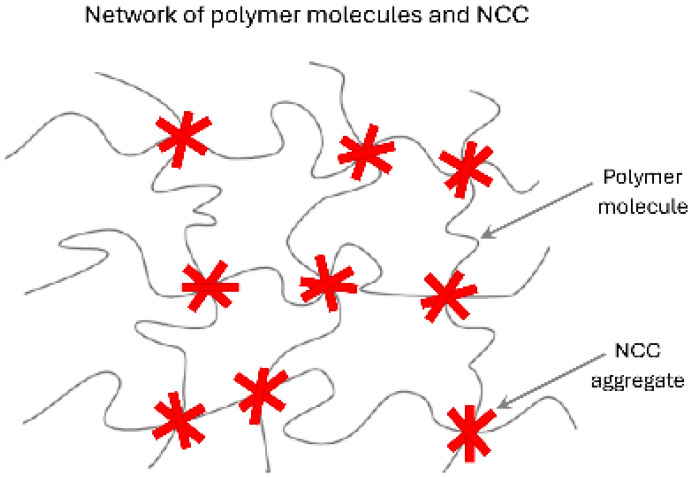
Three-dimensional network structure of nanocrystal aggregates and polymer macromolecules.

The interactions between negatively charged cellulose nanocrystals (NCC) and different polymers are summarized in Table 3. Mixtures of NCC–CMC and NCC–guar gum exhibit strong interactions as reflected in their rheological parameters (K and n). The high values of K and low values of n exhibited by these mixtures are likely to be due to the formation of a three-dimensional interconnected structure of nanocrystal aggregates and polymer macromolecules as depicted schematically in Figure 28.

**Figure 29:**
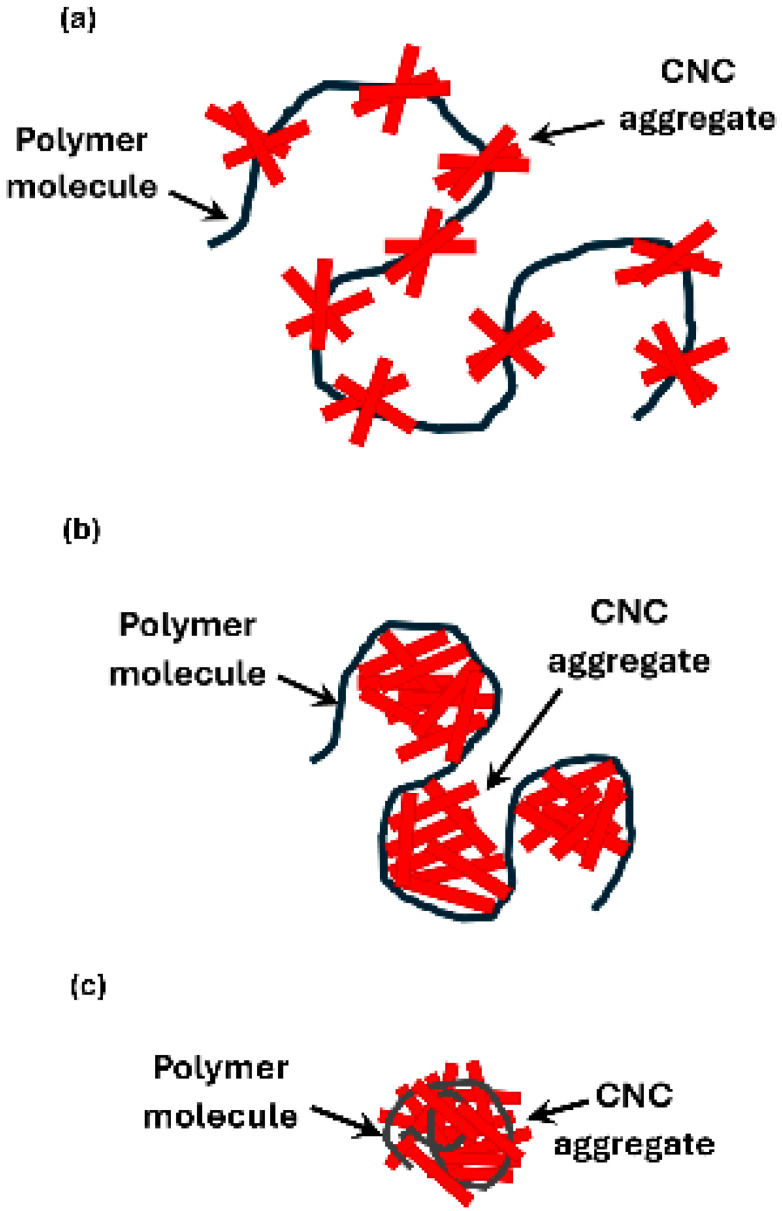
Possible interactions between cellulose nanocrystals (NCC) and polymer chains resulting in a decrease in the consistency of NCC–polymer mixtures.

**Figure 30:**
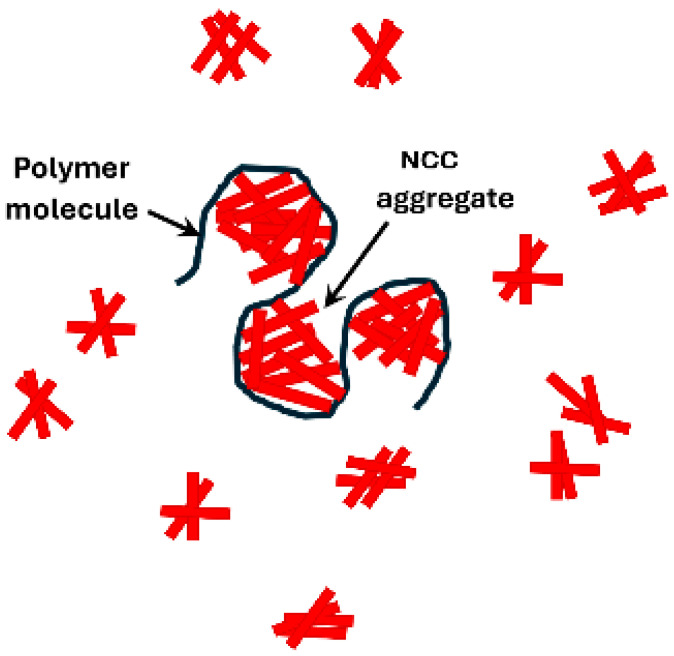
Free NCC aggregates and NCC–polymer aggregates present in NCC–polymer mixtures.

The decrease in consistency index (K) and the observed minimum in K in NCC–WSR-303 mixtures with the addition of cellulose nanocrystals to WSR-303 solution is likely due to the folding or collapse of polymer chains upon the addition of nanocrystals. While the exact nature of interactions between nanocrystals and polymer chains is not known, some possibilities are depicted schematically in Figure 29. In Figure 29a, the nanocrystals form aggregates at various locations on the polymer chain. In Figure 29b, the polymer molecule wraps around the aggregates of cellulose nanocrystals. In Figure 29c, the polymer chains collapse onto the aggregates of cellulose nanocrystals. The folding or collapse of polymer chains can explain the decrease in consistency of NCC–polymer mixtures. In NCC–polymer mixtures where the changes in rheological properties are observed to be negligible (xanthan gum, Praestol 2505, JR-400), it is possible that some of the NCC aggregated with the polymer chains resulting in the folding of chains, and some of the NCC remained in the aqueous phase, as shown schematically in Figure 30. The folding of the polymer chains would tend to decrease the consistency whereas the addition of NCC to the aqueous phase would tend to increase the consistency. Due to these two opposing effects, there occurs negligible changes in the rheological properties of the NCC–polymer mixtures.

Finally, it should be noted that the discussion of NCC–polymer microstructure presented here is somewhat speculative as there is no experimental proof available at present. Clearly further studies are needed to explore the microstructure details of NCC–polymer mixtures experimentally using appropriate imaging techniques. Dynamic measurements of rheology such as storage and loss moduli would also be helpful in further identifying the underlying mechanisms.

## 5. Conclusions

The interactions between the cellulose nanocrystals (referred to as NCC) and the polymers were investigated experimentally using rheological measurements. The polymers studied were anionic sodium carboxymethyl cellulose (CMC), non-ionic guar gum, non-ionic polyethylene oxide (WSR-303), anionic xanthan gum, anionic polyacrylamide (Praestol 2505), and cationic quaternary ammonium salt of hydroxyethyl cellulose (JR-400). Based on experimental work, the following conclusions can be drawn:

- The interaction between cellulose nanocrystals (negatively charged) and anionic polymer CMC is strong. The consistency index increases sharply, and the flow behavior index decreases sharply upon the addition of NCC to CMC solution. The changes in consistency and flow behavior indices are explained in terms of the formation of a three-dimensional network of NCC aggregates and polymer chains.
- The interaction between cellulose nanocrystals (negatively charged) and non-ionic guar gum is also strong. The consistency index rises substantially, and flow behavior index decreases with the addition of NCC to guar gum solution. However, the increase in consistency index observed in NCC–guar gum mixtures with the addition of NCC is less severe as compared with the changes observed in NCC–CMC mixtures.
- The interaction between cellulose nanocrystals (negatively charged) and non-ionic polymer WSR-303 is moderate. However, the interaction is both positive and negative in that the consistency index increases as well as decreases depending upon the polymer and NCC concentrations. At polymer concentrations above 0.75 wt%, the consistency index goes through a minimum upon the addition of NCC.
- The interactions between cellulose nanocrystals (negatively charged) and the following polymers are found to be weak in nature: anionic xanthan gum, anionic Praestol 2505, and cationic JR-400. The changes observed in the consistency and flow behavior indices upon the addition of NCC to polymer solutions are small or negligible.
- It appears that the electric charge on polymers is an important factor in the interaction strength between NCC and various polymers. However, more work needs to be carried out on the mechanisms of interaction to provide a definite answer. The interaction between negatively charged NCC and non-ionic polymers (guar gum and WSR-303) are strong to modest in terms of their impact on the rheological parameters K and n. The interactions between NCC and anionic polymers (xanthan gum and Praestol 2505) are weak due to the presence of the same charge on NCC and polymers. However, anionic CMC and NCC show very strong interactions in terms of their impact on the rheological parameters K and n even though both (CMC and NCC) are of the same charge. The interaction between cationic JR-400 polymer and negatively charged NCC is weak in terms of its impact on the rheological parameters K and n. Clearly, the aggregation of NCC and JR-400 polymer occurs due to the attraction of oppositely charged species. Furthermore, precipitation of NCC–polymer aggregates occur at high concentration of NCC.

Further studies are needed to explore the microstructure details of NCC–polymer mixtures experimentally using appropriate imaging techniques. Dynamic measurements of rheology such as storage and loss moduli would also be helpful in further identifying the underlying mechanisms of interactions between NCC and polymers.

## Figures and Tables

**Table 1:** Different polymers investigated in this work.

Trade Name	Chemical Name, Polymer Type (Ionic or Non-Ionic), and Structure	Industrial Uses
Hercules Cellulose Gum, manufactured by Hercules Inc., Wilmington, DE, USA	Sodium carboxymethyl cellulose (CMC). It is an anionic water-soluble polymer. 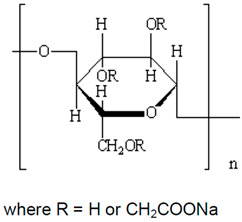	It is used as a thickener, stabilizer, emulsifier, and water retention agent in food products. It is also used as a drug carrier, binder, film-forming material in pharmaceuticals and cosmetics. It is used in drilling fluids and as an anti-coagulant in paper and textile industries. It is also used as a detergent, flocculant, and chelating agent in various industries. In oil and mining industries, it is used as a flotation agent.
Guar Gum, supplied by Sigma-Aldrich, Oakville, ON, Canada	Galactomannan polysaccharide. It is a non-ionic water-soluble polymer. 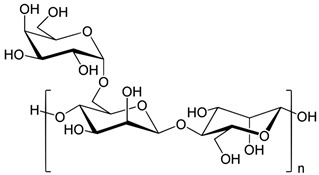	It is a gel-forming agent that can be used for thickening cold and hot liquids, making cottage cheeses, curds, yogurt, sauces, soups, frozen desserts, providing soluble dietary fiber, stabilizing emulsions. It is also used in dairy products, condiments, baked goods, and non-food products.
Polyox WSR-303, ChemPoint, Bellevue, WA, USA	Polyethylene oxide. It is a non-ionic water-soluble polymer. 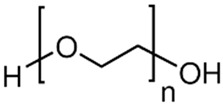	High molecular weight thickener and binder. It is also used as a film former, lubricant, and retention aid. It is used in the manufacturing of ceramics, building and construction materials, lubricants, papermaking, and electronics materials.
Kelzan, manufactured by CP Kelco, Atlanta, GA, USA	Xanthan gum polysaccharide. It is an anionic water-soluble polymer. 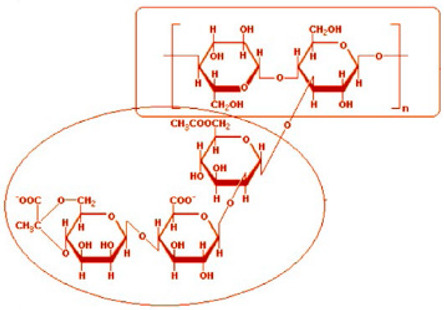	It is a common food additive. It is used as a thickening agent in toothpaste and other industrial products. It improves texture and consistency in ice cream, salad dressings, and baked goods.
Praestol 2505, manufacured by Stcokhausen Inc., Krefeld, Germany.	Polyacrylamide. It is an anionic water-soluble polymer. 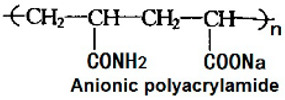	It is a flocculant used in many applications such as dewatering sludges, treating municipal wastewater, and clarifying raw or surface water for drinking water production.
UCARE polymer JR-400, manufactured by Dow chemical company, Midland, MI, USA	Quaternary ammonium salt of hydroxyethyl cellulose. It is a cationic water-soluble polymer. The structure of the repeat unit is as follows: 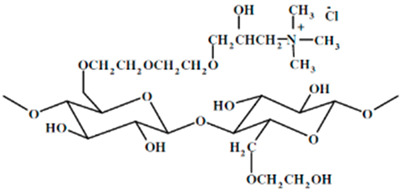	It is used extensively in the preparation of hair and skin care products such as: shampoos, conditioners, body washes, facial cleansers, liquid and bar soaps, moisturizers.

**Table 2:** Relevant dimensions of viscometers used in this study.

Viscometer	$\mathrm{Inner}\;\mathrm{Cylinder}\;\mathrm{Radius},\;R_{i}$	$\mathrm{Outer}\;\mathrm{Cylinder}\;\mathrm{Radius},\;R_{o}$	Length of Inner Cylinder	Gap Width
Fann 35A/SR-12 (low torsion spring constant)	1.72 cm	1.84 cm	3.8 cm	0.12 cm
Fann 35A (high torsion spring constant)	1.72 cm	1.84 cm	3.8 cm	0.12 cm
Haake Roto-visco RV 12 with MV I	2.00 cm	2.1 cm	6.0 cm	0.10 cm
Haake Roto-visco RV 12 with MV II	1.84 cm	2.1 cm	6.0 cm	0.26 cm
Haake Roto-visco RV 12 with MV III	1.52 cm	2.1 cm	6.0 cm	0.58 cm

**Table 3:** Summary of interactions between NCC and polymer.

Polymer	NCC–Polymer Combination. The Sign of Electric Charge Is Indicated as Superscript.	Comments
Anionic (CMC)	NCC^−^ P^−^	Strong interaction observed between negatively charged cellulose nanocrystals and anionic polymer. The consistency index increases sharply with the addition of NCC to CMC solution indicating that the addition of NCC makes the solution much more viscous and thicker. The flow behavior index decreases sharply with the addition of NCC to CMC solution indicating an increase in the degree of shear-thinning. The images of NCC–CMC mixtures show gel-type material at high NCC concentrations.
Non-ionic (Guar Gum)	NCC^−^ P°	Strong interaction observed between negatively charged cellulose nanocrystals and non-ionic polymer. The consistency index increases substantially and the flow behavior index decreases significantly with the addition of NCC to guar gum solution. However, the changes observed in consistency index and flow behavior index with the addition of NCC in NCC–guar gum mixtures are less severe as compared with the changes observed in NCC–CMC mixtures.
Non-ionic (WSR-303)	NCC^−^ P°	Moderate interaction observed between negatively charged cellulose nanocrystals and non-ionic polymer. At low polymer concentrations, the consistency index increases upon addition of NCC. At high polymer concentrations, the consistany index goes through a minimum, that is, it decreases intitially and then rises with the addition of NCC.
Anionic (Xanthan Gum)	NCC^−^ P^−^	Weak interaction observed between negatively charged cellulose nanocrystals and anionic polymer based on consistency index data; consistency index remains nearly constant upon addition of NCC to polymer solution.
Anionic (Praestol 2505)	NCC^−^ P^−^	Weak interaction observed between negatively charged cellulose nanocrystals and anionic polymer. The addition of NCC to polymer has negligible effect on consistency index.
Cationic (JR-400)	NCC^−^ P^+^	Weak interaction observed between negatively charged cellulose nanocrystals and cationic polymer. The addition of NCC to polymer has negligible effect on consistency index. Precipitation of nanocrystals occurs at high concentration of NCC.

## Data Availability

The raw data supporting the conclusions of this article will be made available by the authors on request.
